# Automated closed-loop management of body temperature using forced-air blankets: preliminary feasibility study in a porcine model

**DOI:** 10.1186/s12871-018-0542-4

**Published:** 2018-07-03

**Authors:** Jörg Peter, Kathrin Klingert, Wilfried Klingert, Karolin Thiel, Alfred Königsrainer, Christian Grasshoff, Wolfgang Rosenstiel, Martin Schenk

**Affiliations:** 1 Department of Computer Engineering, University of Tübingen, Sand 13, Tübingen, 72076 Germany; 2 Department of Anaesthesiology and Intensive Care Medicine, University Hospital Tübingen, Hoppe-Seyler-Straße 3, Tübingen, 72076 Germany; 3 Department of General, Visceral and Transplant Surgery, University Hospital Tübingen, Paul-Ehrlich-Straße 36, Tübingen, 72076 Germany; 4 Department of Information Technology and Applied Medical Informatics, University Hospital Tübingen, Sand 13, Tübingen, 72076 Germany

**Keywords:** Targeted temperature management, Forced-air blankets, Warming unit, Closed-loop, Automation, Intensive care, Medical framework, Microcontroller, Patient monitoring

## Abstract

**Background:**

Management of a patient’s body temperature is an important aspect of care that should be addressed by targeted temperature management (TTM). Often, non-invasive methods like forced-air blankets are used. Especially in the operating room this management may be a subsidiary and repetitive task requiring constant observation of the patient’s body temperature and adaption using the limited set of available settings. Thus, automation of TTM is a feasible target to improve patient outcome and reduce caregiver workload.

**Methods:**

A Philips IntelliVue MP 50 patient monitor with an arterial PiCCO catheter system was used to measure patient blood temperature. Thermal management was performed with a 3M Bair Hugger 755 warming unit with forced air blankets. The warming unit was extended by a computer interface to allow for remote and automated control. A proposed closed-loop algorithm reads the measured temperature and performs automated control of the 3M Bair Hugger. Evaluation was performed in an experimental intensive care setting for animal studies. Two fully automated trials are compared with two manual and two uncontrolled trials in the same study setting using six female pigs for prolonged observation times of up to 90 hours in each trial.

**Results:**

The developed system and proposed algorithm allow more precise temperature management by keeping a set target temperature within a range of ± 0.5 °C in 88% of the observation time and within a range of ± 1.0 °C at all times. The proposed algorithm yielded better performance than did manual control or uncontrolled trials. It was able to adapt to individual patient needs as it is more dynamic than look-up table approaches with fixed settings for various temperatures.

**Conclusions:**

Closed-loop TTM using non-invasive forced-air warming blankets was successfully tested in a porcine study with the proposed hardware interface and control algorithm. This automation can be beneficial for patient outcome and can reduce caregiver workload and patient risk in clinical settings. As temperature readings are most often available, existing devices like the 3M Bair Hugger can easily be expanded. However, even if clinical application is feasible, open questions regarding approval and certification of such automated systems within the current legal situation still need to be answered.

**Electronic supplementary material:**

The online version of this article (10.1186/s12871-018-0542-4) contains supplementary material, which is available to authorized users.

## Background

The body’s core temperature is a vital parameter that should normally be kept within the physiological range of 36.0 °C to 37.5 °C (normothermia). Measurements can be performed with a broad variety of methods: oral, rectal or with special catheters in the bladder or the blood vessels [1, 2]. Whereas the body normally is able to maintain normothermia by compensating heat loss through radiation, conduction, convection and evaporation, heat loss during surgery prevails [2]. As soon as the patient is under general anesthesia, medication additionally influences the core body temperature and vasodilatation leads to redistribution of the body heat causing a decrease in body temperature of about 0.5 °C to 1.5 °C within the first 30 minutes [3, 4]. This trend continues in a diminished manner until a new equilibrium is established after three to five hours [4, 5]. Patients with perioperative hypothermia after general anesthesia often complain of hypothermia and nausea [6]. Serious complications of hypothermia are cardiac events like arrhythmia and heart attacks [7], coagulopathy, increased transfusion requirements [8, 9] and even pressure ulcers [10]. Even the duration of anesthetics’ action is extended [11] and, at least for smaller and prolonged surgeries, a higher risk of wound infection is indicated [12]. Other important side-effects of perioperative hypothermia are changes in the potassium serum concentration [10] and reduction of the subcutaneous oxygen partial pressure [3]. Hence it has a crucial influence on the operation’s quality and the postoperative course. Active pre-warming can be used to stabilize the patient during short operations or the initial phase and further managed during prolonged interventions and ICU stays. Nevertheless, excess warming needs to be prevented as elevated body temperature is an important predictor for increased ICU and hospital stay as well as mortality rate [13]. Still, patient temperature is often not regularly checked in the perioperative or ICU setting [14].

Independent of therapeutic goals and the desired target temperature for the patient, measurement and management should be performed regularly in all cases when the patient is under general anesthesia or subsequently recovering in an ICU to ensure patient safety and maintain stable conditions during intensive care. Therefore, therapeutic hypothermia should not be considered a special case but part of targeted temperature management (TTM). It is an active intensive care treatment strategy for attaining and maintaining a desired body temperature as a therapeutic measure for improving patient outcome. This may even be extended to different therapeutic temperature profiles during the course of a therapy [15].

To maintain a desired temperature level during patient care, different more or less invasive methods can be applied. One of the latter are warming pads placed under the patient, especially on the operating table [16]. Another approach is forced-air warming: by laying special air-filled blankets on the patient, a steady stream of air with a predefined temperature on the patient’s skin can be provided [4]. They can be used for cooling or warming and help prevent conductive and convective heat loss [17].

Unfortunately, temperature management with such devices like warming pads and forced-air blankets must currently be performed by a physician or nurse manually. The measured temperature must be kept under observation, and must be adjusted accordingly to maintain the desired patient temperature. The most challenging task is the dynamic adaption to different body heat production rates influencing the patient’s temperature. Thus, simple thresholds and speed settings cannot be used as the required amount of cooling and or warming is dependent on the individual patient and her or his current condition. This makes management of patient temperature, among other jobs, a good example of tasks still requiring manual observation and adaption according to simple goal-directed rules of medical practice.

To address automation for temperature management, we developed a novel approach for closed-loop control of patient body temperature using a modified forced-air patient warming device and temperature readings from a patient monitor to automatically adapt the body to a predefined temperature and perform TTM.

Beyond TTM, the development and evaluation of closed-loop systems for perioperative and intensive care is an important and ongoing field of research, including manifold applications like goal-directed fluid management, anesthesia and analgesia or automated homeostasis management. By using closed-loop systems for such manual tasks, workload and human error potential can be reduced as successfully shown in other examples like automated anticoagulation management [18].

Given this broad range of closed-loop control applications, the presented TTM system is itself part of an extended ICU automation system, using closed-loop control for various vital parameters to maintain homeostasis in a porcine model [19] and evaluating the automation of different detection algorithms such as for the detection of blood withdrawal by characteristic changes in vital parameters [20].

## Methods

**Fig. 1:**
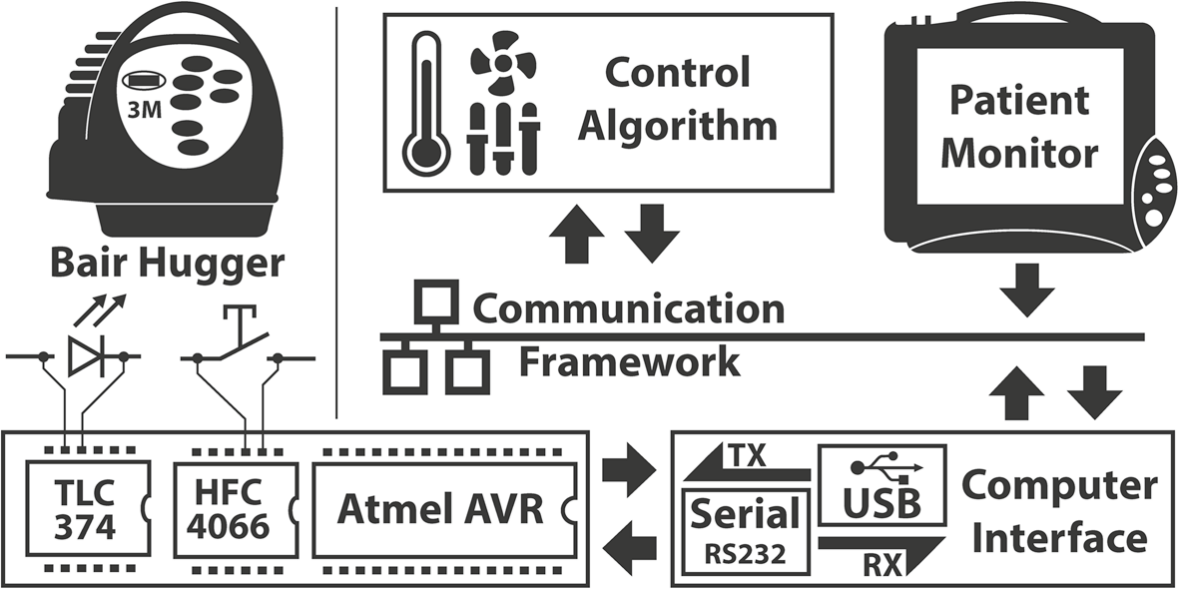
Schematic setup and data flow. Illustration of the data access and processing for remote control of the 3M Bair Hugger warming device

The general setup consisted of a 3M Bair Hugger 755 [21] temperature management unit for temperature control and a Philips IntelliVue MP50 patient monitor for obtaining patient temperature readings. The devices are interconnected with each other in an experimental clinical measurement and control framework (TICoMS) [22]. For remote control of the 3M Bair Hugger an add-on board, connected to a computer by a USB to serial converter, was developed. The temperature readings were processed with a proposed control algorithm and control commands were sent to the 3M Bair Hugger via the developed interface. The entire setup is visualized in Fig. 1.

Development of the device interfaces and the algorithm for temperature management was performed in several steps: In a first step, the 3M Bair Hugger’s electronic circuit boards were analyzed and a hardware interface for reading the current operational state and remote control was developed. The next step consisted of integrating the hardware in TICoMS and implementing the control algorithm for automated closed-loop control using temperature readings from the Philips patient monitor. The algorithm itself and the steps required for implementation and evaluation are presented in the subsequent sections.

### Bair Hugger

The used 3M Bair Hugger 755 warming unit [21] device allows three predefined temperature levels to be set, 32±1.5 °C, 38±1.5 °C and 43±1.5 °C, and ambient temperature (about 21 °C in the used setting). The airflow speed can be adapted with two speed settings: *fast* (4700 rpm) or *slow* (4100 rpm), resulting in a maximal airflow of 23*L*/*s* [21]. To obtain access to those settings, a hardware interface was developed.

### Hardware interface

The interface for the 3M Bair Hugger 755 was implemented with a custom circuit board, intercepting communication with the 3M Bair Hugger’s display circuit board, simulating key presses and communicating with a computer using an Atmel AVR Mega8A [23] (Microchip Technology Inc.) microcontroller. It is designed and implemented to work alongside manual control of the 3M Bair Hugger, which provided a safety override for the automated commands. The Atmel Mega8A microcontroller was used, as it provided a serial communication interface and the required number of input and output pins to obtain the device’s states and address the switches. The information given on the lit LEDs, indicating the states of the 3M Bair Hugger device, was intercepted using TLC 374 comparator ICs and fed to the input pins of the microcontroller. The available LEDs indicate a fault state and temperature in range as well as settings for the operation modes: standby, slow speed, fast speed and settings for the temperature: ambient, 32 °C, 38 °C, 43 °C [21]. In total ten input pins were used to read those states from the indicator LEDs. Simulation of key strokes on the 3M Bair Hugger was performed with HFC 4066 ICs as digital switches. These were connected in parallel to the manual switches that can be pressed on the front of the device. In total, eight output pins were used to control the 3M Bair Hugger switches and preliminary tests indicated that a press time of 150 ms is a reliable time for detection by the 3M Bair Hugger circuit.

**Fig. 2:**
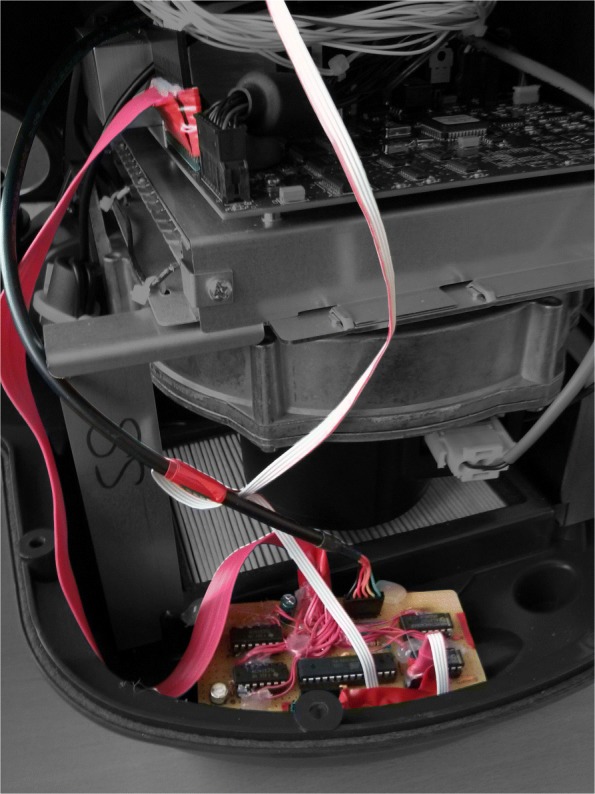
Add-on board integration to 3M Bair Hugger. Image of the opened 3M Bair Hugger device with the installed add-on board for reading the current device state and remote control of the switches. Original 3M Bair Hugger parts, desaturated in color image

Information regarding the current device state is sent once a second and control commands can be applied to the 3M Bair Hugger via a bidirectional serial communication channel. To obtain stable serial communication with a speed of 9600 Baud, a matched quartz crystal with 15.7456 *MHz* as the operation frequency of the microcontroller was used. For connection of the serial interface of the microcontroller to a computer a serial to USB adapter (Delock 83784 with FTDI 232RL Chip) was used. Integration of the developed add-on board into the Bair Hugger unit is shown in Fig. 2. For schematics see Additional file 1.

#### Controller software and serial protocol

The software of the Atmel Mega 8A 8-bit microcontroller was implemented in C using Atmel Studio 6 with GCC [see Additional file 2]. Interrupts were used for a timer to read and send the current state of the 3M Bair Hugger obtained from the LEDs once a second and to apply received serial control commands by pressing the switches. The serial communication protocol was kept simple for the sake of proving the concept of automation. Each message is transmitted in alphanumerical ASCII format and framed with a start and end character: 0x02 (STX) and 0x03 (ETX), respectively. The information given by the 3M Bair Hugger’s LED states is sent as a numerical string of 0s and 1s indicating the respective states of the LEDs. Switches on the 3M Bair Hugger can be remotely operated by sending a command like S:X to the microcontroller, where X is the number of the Switch (1-8) that should be set.

### Control algorithm

Temperature management is performed by adapting the 3M Bair Hugger temperature setting, depending on the measured patient temperature, using the closed-loop algorithm presented below. For reduction of interferences and disturbances in the temperature reading, measured data are collected and averaged over a short time frame of a few minutes. Thereafter, the averaged value is used to adapt the temperature setting.

The proposed algorithm uses three parameters: an evaluation interval for the adaption *c*_*t*_, a target temperature *T*_*tar*_ and a scaling factor *c*_*s*_ for the power of adaption. This scaling factor allows the feedback control system to be adapted to a specific clinical setting, thus facilitating a sufficiently fast response while prohibiting overshoot. The current temperature for each control step is calculated with all collected measurements within the observation time frame by using the arithmetic mean. For this, the current point in time *t*, the fixed interval *c*_*t*_ in seconds, the target temperature *T*_*tar*_ and the current body temperature *T*_*t*_ at time *t* are used:

$$\overline{T_{t}} = \frac{1}{c_{t}} \cdot \sum_{i=t-c_{t}}^{t} T_{i} $$

Next, the difference between the currently observed measurement $\overline {T_{t}}$ and the target temperature is calculated:

$$\Delta_{t} = \overline{T_{t}} -T_{tar} $$

Then, a state *s*_*t*_∈[−1,1] at time *t* is calculated using the desired target *Δ*_*t*_ and the scaling factor *c*_*s*_. This state is limited to a desired interval [−1,1]. This state variable *s*_*t*_ represents the current action, whereas *s*=−1 means maximal cooling and *s*=+1 means maximal heating. For *t*=0 this is initially set to zero (*s*_0_=0) as a central starting point for the dynamic algorithm.

$$\begin{aligned} s_{t} = \left\{ \begin{array}{rlrrr} \quad 1 & \text{~~~if~~} & & \Delta_{t} \cdot c_{s} \geq & 1\\ \Delta_{t} \cdot c_{s} & \text{~~~if~~} & -1 < & s_{t} < & 1 \\ -1 & \text{~~~if~~} & & \Delta_{t} \cdot c_{s} \leq &-1\\ \end{array}\right. \end{aligned} $$

In a last step, the device settings need to be chosen according to the control state *s*_*t*_. The device setting is a tuple consisting of speed and heat setting *x*_*t*_=(*speed*,*heat*) with *speed*∈{*standby*,*slow*,*fast*} and *heat*∈{21 °C,32 °C,38 °C,43 °C}. The *standby* speed setting is not used for the control, as this would deflate the forced-air blanket and therefore is impractical. The setting for ambient temperature is denoted as 21 °C, as this is a common room temperature and present in the used setting.

$$\begin{aligned} x_{t} = \left\{ \begin{array}{lllll} (21\ ^\circ\text{C},fast) &\quad \text{if} & & \, \, \, s_{t} = & \quad -1\\ (21\ ^\circ\text{C},slow) &\quad \text{if} & \, \, \, -1< & \, \, \, s_{t}\leq & \, \, -0.5 \\ (32\, ^\circ\text{C},slow) &\quad \text{if} & \, \, \, 0.5< & \, \, \, s_{t}\leq & \quad\,\,\ \ 0\\ (38\ ^\circ\text{C},slow) &\quad \text{if} & \quad\, 0< & \, \, \, s_{t}\leq & \quad\, 0.5\\ (43\ ^\circ\text{C},slow) &\quad \text{if} & \,\,\, 0.5< & \, \, \, s_{t}< & \quad\ \ \ \, 1\\ (43\ ^\circ\text{C},fast) &\quad \text{if} & & \, \, \, s_{t}= & \quad\ \ \ \, 1\\ \end{array}\right. \end{aligned} $$

The calculated tuple *x*_*t*_ is then used to set the 3M Bair Hugger device via the developed interface board. Processing is repeated if the defined time interval *c*_*t*_ is reached again.

### Framework integration and implementation

The modified 3M Bair Hugger device with the developed add-on board was connected to a computer and integrated into the TICoMS medical framework [22] used for monitoring and controlling many other medical devices in the used experimental ICU setup. All communication within the framework is performed by XML-based messages. Monitoring information and control commands from all devices are stored in a central PostgreSQL study database. Temperature management was integrated by implementing two C++/Qt application plugins. A first one is used to integrate the 3M Bair Hugger device into the framework by establishing the serial communication to the 3M Bair Hugger with the developed add-on board. Current settings are pushed to the framework as messages and control messages are processed and executed. The second application plugin implements the proposed algorithm and obtains temperature readings through messages. The required temperature readings are obtained as messages from another plugin implementing the *Philips IntelliVue* communication protocol for communication with the Philips MP50 monitor. The received data is evaluated with the algorithm and control commands are sent to the first described application for communication with the 3M Bair Hugger add-on board. In addition to those two essential plugins for automated control of the 3M Bair Hugger, a graphical user interface (GUI) on a central touchscreen interface was implemented for interaction and manual temperature control.

### Evaluation

Evaluation of the proposed hardware and algorithm was performed during ongoing animal studies to identify parameters for fluid responsiveness and closed-loop homoeostasis management. The studies were performed with domesticated, female pigs (German Landrace) in an experimental porcine ICU setting at the University Hospital of Tübingen. Those studies were approved by the local Animal Experiment Committee in accordance with the National Guidelines for Animal Care and Handling.

#### Animal preparation

Animal preparation was performed as in previous and related studies [19]. The animals were housed on straw in a 6 *m*^2^ cage, were fed with standard diet and had free access to water. In preparation for the study, the pigs were fasted overnight, given free access to water and passed through premedication under intramuscular atropine 0.05 mg/kg and azaperone 2-4 mg/kg in a cervical neck muscle. Anesthesia was induced ten minutes after premedication with intramuscular midazolam 0.5-2 mg/kg and ketamine 14 mg/kg. As soon as the pigs were asleep, a peripheral venous catheter was placed in one of the ear veins. Through this access propofol 2 mg/kg was administered intravenously. Thereafter, the trachea was intubated with a 6.5-7.5F endotracheal tube and a gastric tube was placed by a vet.

To gain access to arteries and veins, a five-lumen central venous catheter was placed in the external jugular vein and an arterial catheter (PiCCO system) was inserted into the femoral artery. Subsequently, a suprapubic catheter was placed in the bladder. All three placements were performed with ultrasound guidance.

During the study ventilation was performed with a volume-controlled mode with a tidal volume of 10 mL/kg. A positive end-expiratory pressure (PEEP) of 5 mmHg was chosen to prevent atelectasis, and the *FiO*_2_ in the gas mixture was set at 0.40 on the medical ventilator (Dräger Evita XL).

To prevent any pain or suffering, general anesthesia was then maintained by administering total intravenous anesthesia (TIVA) consisting of ketamine (15 mg/kg/h), fentanyl (20 *μ*g/kg/h) and midazolam (0.9 mg/kg/h) for the entire duration of the study. After completion of the medical study, the animals were sacrificed in deep anesthesia with an intravenous injection of embutramide (T61).

#### Performance assessment

Assessment of the algorithm’s performance was performed in comparison to trials performed without a forced-air blanket and trials with manual control of the 3M Bair Hugger device. These three observation groups for temperature management are denoted *no control* (N), *manual* (M) and *automated* (A) and each consists of two trials. All six female pigs were about 3 months old and had average weights of 43.6 ±2.9 *SEM* (N group), 45.3±3.3 *SEM* (M group) and 40.2±1.2 *SEM* (A group).

For this research 3M lower-body warming blankets (model 525) were used. However, for the used pig model, the blankets covered the entire body of the research animals in the used study setting, providing an ambient temperature of 21 °C. To ensure animal safety during the performed trials, human supervision was given at all times and the patient monitor was configured to output alarms if critical hypo- or hyperthermia was imminent. No further safety measures regarding device malfunction were implemented for this proof-of-concept study.

The target temperature was chosen at 38.0 °C for both manual controlled (M) and automated (A) trials. In the uncontrolled trials (N) no active temperature management using forced-air blankets was performed. As pigs have a slightly increased normal temperature in comparison to humans, the chosen target temperature was normothermic. Each trial had an approved and planned duration of 90 hours. Configuration of the automated closed-loop algorithm was performed by setting target temperature *T*_*tar*_=38.0 °C, evaluation time *c*_*t*_=120 *s*, and scaling factor *c*_*s*_=0.7. This scaling was empirically chosen to fit the used porcine model, room temperature and application of the warming blankets.

For comparison of the results, the performance metric for how well the temperature could be kept at the desired target temperature within a temperature range of ± 0.5 °C and ± 1.0 °C for the whole duration of each trial in three study conditions was chosen. Temperature readings were obtained with the Philips MP50 monitor from arterial temperature measures using a connected PiCCO catheter system (MAQUET Holding B.V. & Co. KG, Germany).

## Results

**Fig. 3:**
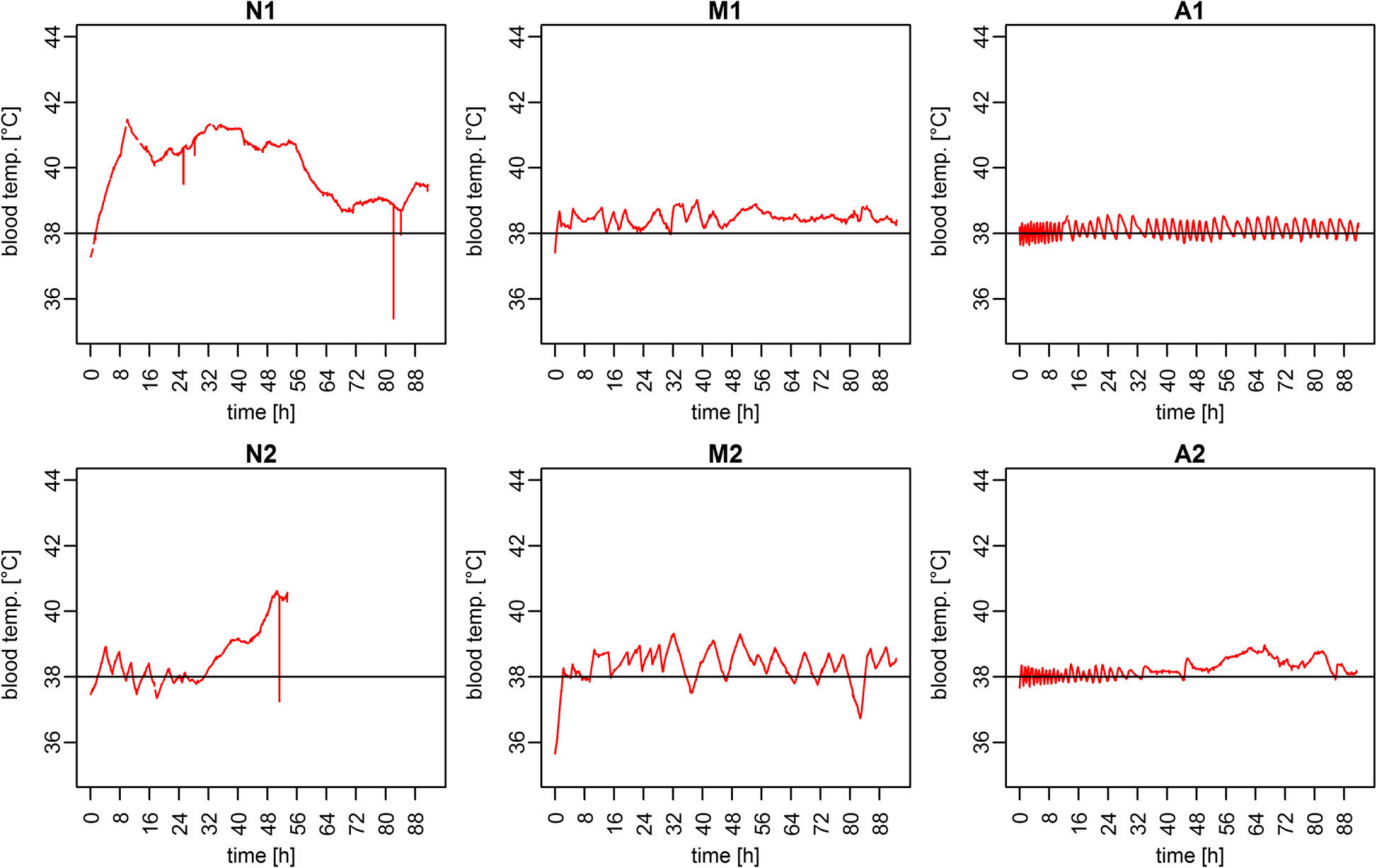
Measured blood temperatures. Measurements of the blood temperatures obtained from the patient monitor for each two trials of the three different groups (visualized in rows) with a reference line at the normal and target temperature of 38 °C

**Fig. 4:**
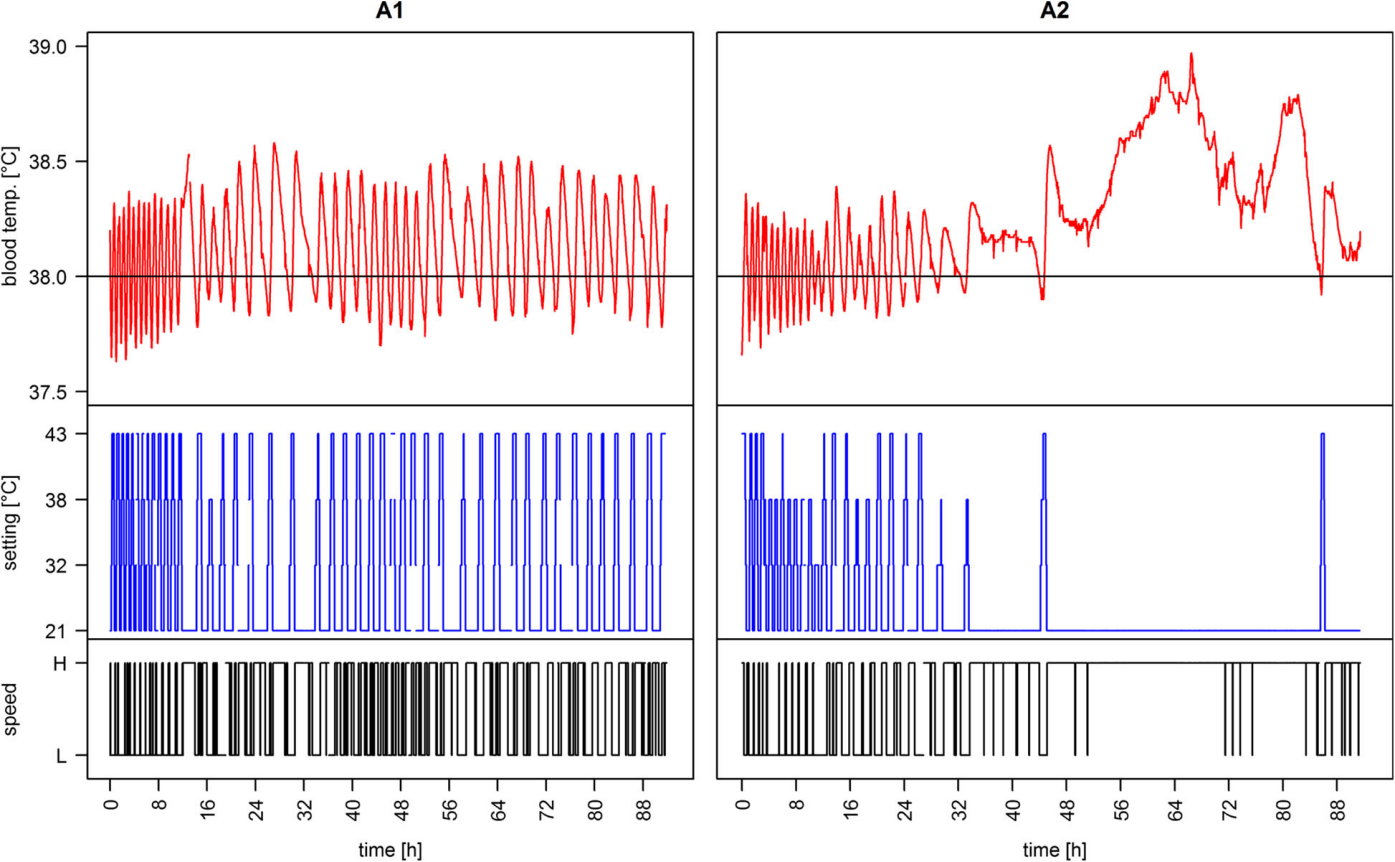
Detail visualization of automated trials. Detailed time series of the device settings for temperature and speed and the corresponding changes in blood temperature for the two automated trials

The proposed hardware solution for interacting with the 3M Bair Hugger temperature management unit via the developed add-on board and the proposed control algorithm was successfully tested in two automated trials of an ongoing animal study. The data collected from patient monitor and 3M Bair Hugger were successfully stored in the study database [Results in Additional file 3]. Observed blood temperatures for the individual trials in all three study conditions are shown in Fig. 3 as median values with a sliding window of *c*_*t*_=120 *s* for filtering short interruptions due to flushing or manual infusions with cold fluids. It can clearly be observed that the animals in the uncontrolled trials N1 and N2 developed hyperthermia. Manual control during trials M1 and M2 provided adequate temperature management at the desired target temperature of 38.0 °C. This can also be observed for the automated trials A1 and A2. Additionally, the closer adaption intervals in the automated trials (A), in comparison to the manually controlled trials (M) with a tighter temperature control, can be seen. For better visualization of the algorithmic adaption, the results of the automatically controlled trials (A) are presented in Fig. 4. Each of the temperatures measured during the study duration of about 90 hours are plotted alongside the corresponding settings for the speed and temperature of the 3M Bair Hugger device. It can be observed that individual adaptions were necessary for the two animals to maintain the set target temperature. Especially in trial A1, small oscillations caused by the tight temperature control of the algorithm can be seen. For evaluation of performance, the percentage of time during which temperature remained within the ranges of ± 0.5 °C and ± 1.0 °C around the target temperature was evaluated using the total duration of the individual trial as a reference and comparing it to the temperature measurements obtained from the patient monitor once a second. The results of all trials of the three observation groups are presented in Table 1. The uncontrolled trial N2 was unfortunately cut short by medical conditions unrelated to temperature management (pneumothorax after catheter placement). This resulted in a shorter observation time for evaluation of the temperature in this reference trial. The number of adaptions made in the individual trials is shown in Table 2. It can be observed that the number of adaptions made by the automated system (A) is significantly larger than for the manual group (M).

**Table 1:** Performance results for the individual trials

Trial		N1	N2	M1	M2	A1	A2
Total	[*s*]	330060	192168	333490	333020	330960	329270
Target range ± 0.5 °C							
Outside	[*s*]	302527	82947	127111	159037	5883	73330
Within	[*%*]	8.34	56.85	61.88	52.24	98.22	77.73
Target range ± 1.0 °C							
Outside	[*s*]	243583	55522	579	23538	0	0
Within	[*%*]	26.20	71.11	99.83	92.93	100.00	100.00

Shown are the total duration of the individual trials and the times exceeding the target temperature by ± 0.5 °*C* and ± 1.0 °*C* in seconds. Overall performance for the two evaluated temperature ranges is shown below

**Table 2:** Total number of performed temperature adaptions and relative to trial duration

Trial	N1	N2	M1	M2	A1	A2
Num. of changes	–	–	78	58	443	215
Changes per hour	–	–	0.84	0.63	4.82	2.35

For the unmanaged group (N), no forced-air temperature management device was used and no adaptions were performed

On average, temperatures for the three observed groups were within the desired target range of 38±0.5 °C (± 1.0 °C) for the following trial duration percentages: no control (N) 32.60% (48.66%), manual (M) 57.06% (96.38%), and automated (A) 87.98% (100.00%). For the ± 1.0 °C range, the temperatures measured for the proposed automated solution were within the target temperatures at all times, closely followed by manual management (96.38% of trial duration). For the stricter temperature constraint of 38°±0.5 °C, the performance gap between manual and automated control increased.

**Fig. 5:**
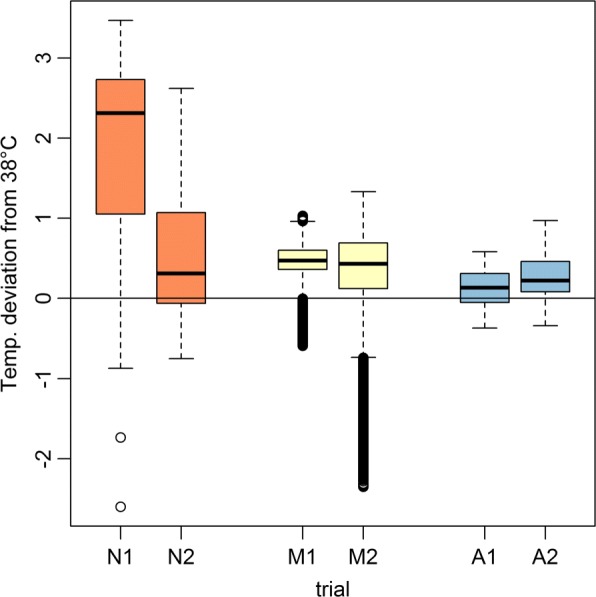
Boxplots of observed deviations from target temperature. Boxplots for visualization of the temperature deviations of the three observed groups: non-controlled (N), manual (M) and automated (A). The desired target temperature of 38 °C is plotted as a reference line. For the uncontrolled trials (N), no temperature management was performed to achieve the target temperature

For better visualization of the results in relation to the set target temperature, the measured temperatures (120 seconds, sliding median) are shown in Fig. 5 for the individual trials plotted as the deviation from the target temperature of 38.0 °C. The uncontrolled trials (N) show significantly increased body temperatures and fever, which was inhibited by temperature management in the manual and automated trials. In all trials the average measured temperature was above the set target temperature.

## Discussion

The aim of this study was to provide proof of concept for automated temperature management using forced-air blankets with a proposed algorithm and to perform a fundamental comparison with uncontrolled and manual trials within the same experimental setting for animal studies. This automation was motivated by the hope that caregiver workload would be decreased and patient outcome improved by reducing patient risk using tight control with an additional reduction in human error potential and variance. By keeping the temperature at the chosen target temperature of 38 °±1.0 °C at all times and within the the 38 °±0.5 °C range for 88 percent of the time, the basic feasibility of this closed-loop approach is successfully shown for the given porcine study model.

The proposed algorithm conducts management mainly by changing temperature, whereas changes in speed settings are performed only in extreme conditions at the boundaries. This behavior was preferred after short preliminary studies indicated the impact between the slow and the fast speed setting to be minor in comparison to the temperature setting.The manufacturer’s data sheet reveals the speed settings to be 4700 rpm and 4100 rpm [21] for the fast and the slow settings, which relates to a flow of 23 L/s and 20 L/s, respectively, thus supporting this observation and decision.

Algorithmic response of the proposed solution is very dynamic as the individual patient’s needs are highly adaptive and can change with time during general anesthesia and prolonged operation periods [3–5]. Restriction to fixed speed and temperature settings in a look-up table approach was therefore not applicable.

The algorithm was designed to anneal to the optimal or at least the best possible setting. Even a slight change in any direction allows rapid adaption of the device. This prohibits locking scenarios in extreme settings that would need a significant amount of time to return from an extreme state, e.g. still heating the patient when cooling is needed. As the individual settings of the used device are limited, adaption cannot be perfect in some cases because no optimal setting may be available for maintaining the target temperature. Thus, alternation between two available settings, where the first one leads to a slight chilling of the patient and the second one to heating, may occur. This device limitation can be observed clearly in the A1 trial shown in Fig. 4. The observable small ramps indicate that no optimal setting was possible and a drift occurred. This led to a change in the settings, resulting in a drift in the opposing direction, which was automatically corrected by reversing those changes again. Such inaccuracies are inevitable unless algorithmic response time is drastically decreased to reduce the effect. However, such alternations may introduce other, maybe more severe problems as they include effects that were specially considered and addressed: increasing device wear, interspersing measurement inaccuracies, or passing through short-term effects like flushing or injections by limiting the averaging of the individual measurements.

In addition to evaluation of absolute performance in relation to the target temperature, a relative comparison to uncontrolled and manual trials was performed. The trials performed with manual control are, of course, a highly subjective matter and no direct or objective relation to the uncontrolled and automated trial can be made as interaction with the 3M Bair Hugger was dependent on the workload and observation of the measured temperature by the individual caregiver. However, as the trials were performed on a single pig each time, individual care like in an operating room setting with good observation and fast reaction times was achieved, and the results of the automated trial show the feasibility of the proposed solution on its own and are consistent with the manually controlled reference.

As the development and evaluation of the automated control system was performed as part of an ongoing study, the number of trials for this first feasibility analysis was limited to two per observation group. However, as the individual trials were observed with a duration of up to 90 hours in a postoperative setting, a variety of metabolic and hemostatic variations could already be covered. For transfer of those results to humans, the superior skin insulation of swine in comparison to that of humans needs to be considered as it may influence performance. This may be addressed by adapting the algorithmic scaling factor and response time.

The occurrence of elevated temperatures in long-term narcosis is a recurring complication in the used porcine model. It may be caused by medication-induced hyperthermia (e.g. by ketamine). For external temperature control, this can be considered an incidental model of fever. For the uncontrolled trials (N1 and N2), this resulted in the observed temperature increase. The rather large performance gap between N1 and N2 can be attributed mainly to the shorter observation period where the fever was just developing. A longer observation time would most likely have led to a performance decline comparable to that of N1. Using temperature management in the manual (M) and automated (A) trials, this effect was able to be compensated by the algorithm and the used forced-air temperature management device during the observation time.

As the time needed for premedication and catheter placement varied between the individual animal subjects, heat loss increased with the preparation time. Especially in animal subject M2, it became obvious that heat transfer between warming pad and animal was not sufficient on the operating table, resulting in minor hypothermia prior to the beginning of automated temperature management.

With regard to the number of temperature changes performed, the automated system was significantly more agile than was manual adaption. This is mainly due to the short control interval of 2 minutes. In addition, the algorithm is designed to perform an iterative adjustment in several steps to achieve the maximum or minimum settings, resulting in an increased number of adaptions. For the automated system the number of adaptions equals roughly two to five changes per hour, whereas for the manual group adaptions were made on average at least every two hours. The difference between the number of changes in A1 and A2 can be explained by the temperature setting. As A2 developed hyperthermia, cooling was performed at the maximum rate most of the time. Thus, no further changes in the settings were made by the algorithm as the best possible setting was already implemented.

However, despite the positive performance indications arising from this feasibility study, several limitations and aspects for further evaluation and a possible clinical application for the future have to be discussed:

First, the thermodynamic aspects of each individual patient may have to be addressed in more detail. Especially body height and weight and the derived BMI significantly influence the heat capacity of the body. Another edge case would be application in pediatric patients, who have a much larger body surface in relation to body mass. Such edge cases were not evaluated in this first feasibility study with a porcine model. Further evaluation of such scenarios should be performed. Yet given the algorithm’s design, a successful dynamic adaption using the closed-loop feedback seems feasible when using temperature annealing. Nevertheless, parameters might have to be adapted to avoid overshooting and temperature oscillation in those scenarios. Additionally, other variations within the clinical workflow, like pre-warming of patients before the introduction of anesthesia, may have to be addressed. As the algorithm tries to anneal to the set target temperature, applied pre-warming might be reduced if the target temperature level is set to a normothermic level. Thus, in such a case, also the target temperature should be raised to maintain the pre-warming temperature or extended temperature profiles, and higher temperatures at the beginning of the therapy should be considered.

Another aspect to be further evaluated may be the use of different types of forced-air warming blankets as for this study the entire body of the study animals was covered. Yet, in clinical application often only upper or lower body blankets are used. Even in this case, the proposed algorithm should also be applicable, provided that heat transfer capacity with the reduced blanket size is still sufficient. However, regardless of feasibility aspect, excessive chilling or heating of limited body surface areas with possible clinical complications would have to be considered and further evaluated in additional studies.

As arterial monitoring was performed with a PiCCO catheter system, interruptions and interference with temperature measurement had to be considered. Disturbances caused by injection of cooled fluid for recalibration of cardiac output every few hours and flushing events were addressed using the averaging filter. However, given scenarios where injection of larger amounts of cold fluid is performed, temperature readings from this source may be impaired.

Therefore, the proposed solution is not limited to measurement by this means but other often used sources like sensors integrated in bladder catheters [1, 2] can easily be used as the algorithm has no fixed configuration for a specific sensor, but performs iterative annealing. For application of the algorithm only temperature offsets between different sensors and adaptions of the averaging time and control strength would need to be considered and evaluated. Regarding the fact that the temperature observed in the performed studies was always slightly above the desired target temperature, such sensor offsets may be considered in the current setup, too. This could be countered in further versions by introducing an additional offset variable into the algorithm. Of course, potential drawbacks of such alternative temperature sources would have to be carefully evaluated. When using bladder temperature, measurements can be impaired by the heat capacity of the fluid and thus lag behind the body’s actual core temperature.

Given the current limitations, there is still much potential for improvement. Temperature control may be improved using machine learning techniques or by including other vital signs and patient data, like body size or weight, to account for their possible impact on the algorithm’s performance. This may allow better, personalized adjustment of the algorithm. Given multiple temperature sensors, redundancy could be established and other calculations like the difference between such temperature readings or delays as indicators for the body’s heat capacity could be used as additional parameters for fine-tuning the algorithmic response. With such an algorithmic solution, control is of course not limited to a single target temperature, but can be used to apply complex therapeutic temperature profiles for TTM [15]. Such temperature profiles with individual times for defined temperatures and temperature ramps could easily be integrated into an extended version.

For evaluation purposes, the circuit developed for this study was limited to fundamental and rudimentary implementation without taking further steps for electrical shielding or decoupling of the signals (e.g., with opto-isolators) to provide isolation for external and internal electrical interference. Only a simple communication protocol without check-sums or error correction was used. These are, of course, critical steps for patient safety as well as for approval of such devices in real clinical environments and certification as a medical product. However, as this research aimed to prove feasibility and not to develop a new certified medical product, such tasks could and should be performed by manufacturers who integrate such capabilities into already existing devices without the need for much additional hardware or modifications.

## Conclusion

In the presented animal study we were able to show that closed-loop temperature management is a feasible method with promising results. As patient monitoring solutions are already present in intensive care or operating room settings, patient temperature readings should be readily available from a broad variety of sources. Additionally, forced-air warming with special air-filled blankets is already an established solution for countering conductive and convective heat loss [17]. Nevertheless, whereas body temperature is an important parameter for patient outcome, temperature management is often performed as a subsidiary and not regularly performed task in perioperative and intensive care [14]. Closed-loop control allows tighter temperature management, faster adaption times, less variance in the patient’s body temperature since human observation and capacities are limited and manual adjustment of the blanket’s temperature each couple of minutes would not be a realistic option. Without the requirement for constant human supervision, automated control for warming blankets might even be used in settings like hospital wards where such monitoring is not guaranteed at all times.

The presented approach uses automated temperature control and provides the concept for a feasible and non-invasive means of temperature management to increase patient safety, improve patient outcome, and reduce caregiver workload. As our goal was to provide a first feasibility analysis for closed-loop temperature control using non-invasive forced-air blankets in an experimental setting and not to develop a new, certified medical product, our hard- and software was a minimal implementation. This aspect would therefore need improvement and additional safety measures for approval. Indeed, such tasks could and should be performed by manufacturers by including the pertinent changes in the already existing monitoring and temperature management devices

However, this is just one of many possible examples for application of closed-loop management in an existing and further developing research area that is not limited to TTM but also covers the multitude of other aspects in intensive care medicine like anesthesia, analgesia and fluid management. Unfortunately, the entire aspect of automating medical procedures, closed-loops and other methods is often very experimental and must still come of age for widespread practical applications. Many questions regarding liability, performance, or ethical decision-making, remain unanswered. This is not at all limited to the medical sector, but also affects other industrial fields and society in general. Experimental studies and modifications of existing medical devices can therefore show feasibility and possible advantages of such automated systems in clinical practice. Still, fundamental automation questions need to be solved in a general context for clinical approval and certification, thus limiting the manufacturing and implementation of such devices during the near future.

## Additional files

Additional file 1 Interface schematics. The additional file interface-schematic.pdf contains full electronic schematics for the presented hardware interface to the 3M Bair Hugger 755. (PDF 84.1 kb)

Additional file 2 Source code for AVR microcontroller. The additional file avr-code.c contains the source code for the used Atmel Mega8A microcontroller. (C 5.81 kb)

Additional file 3 Measurement dataset. The additional file dataset.csv contains the dataset consisting of the temperature readings from the Philips MP 50 patient monitor and the settings for the 3M Bair Hugger for automated (A) controlled trials with one second interval. (CSV 14336 kb)

## Abbreviations

BMI: Body mass index
IC: Integrated circuit
ICU: Intensive care unit
LED: Light emitting diode
OR: Operating room
SEM: Standard error of the mean
TICoMS: Tübinger ICU control and monitoring system
TTM: Targeted temperature management
USB: Universal serial bus
