# Supplementary figures and images for: Automated closed-loop management of body temperature using forced-air blankets: preliminary feasibility study in a porcine model
Source: BMC Anesthesiol. 2018 Jul 3;18:80. doi: 10.1186/s12871-018-0542-4 (PMC6029032; doi:10.1186/s12871-018-0542-4)

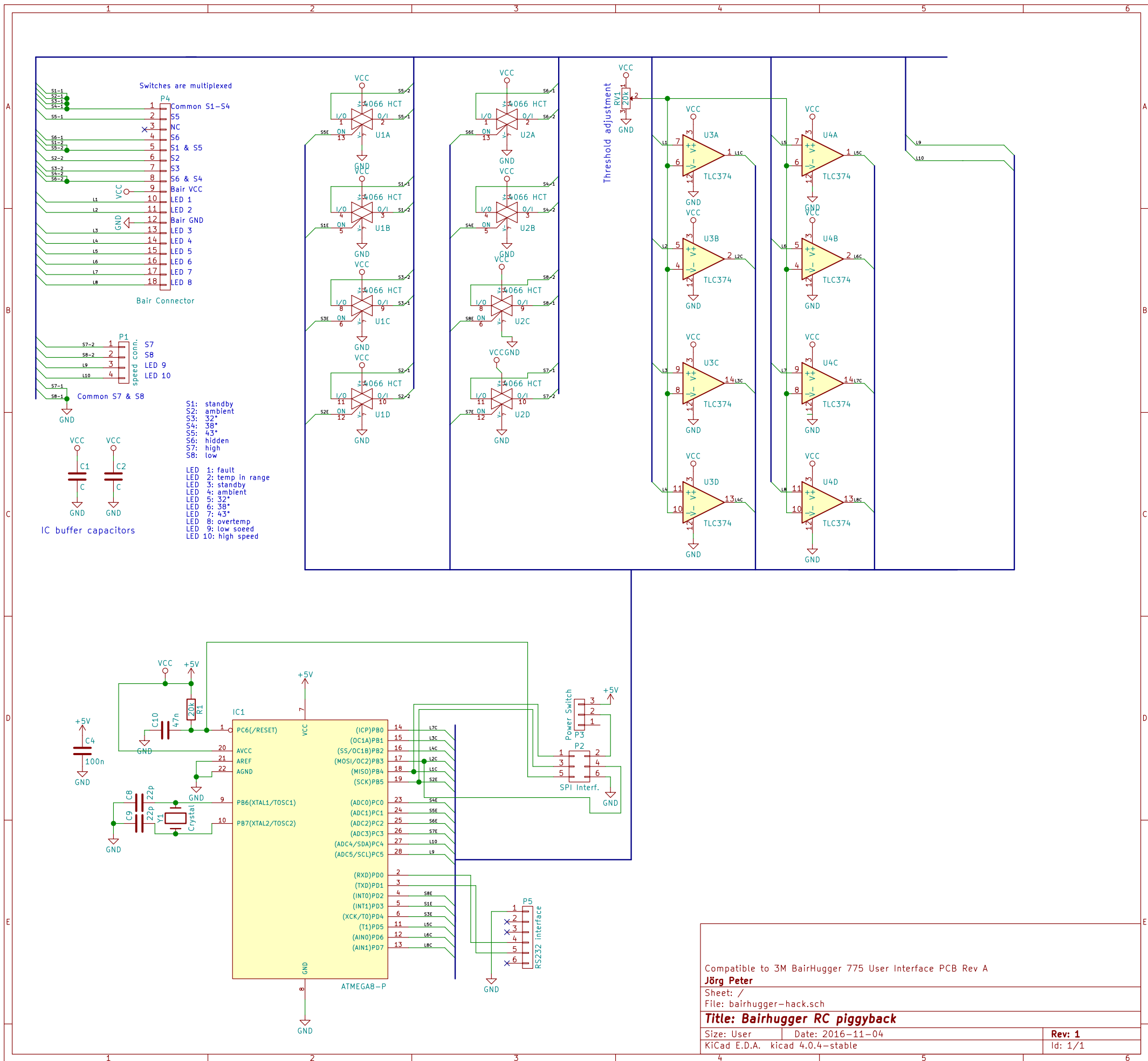

Supplement: Supplementary file 1 — Interface schematics. The additional file interface-schematic.pdf contains full electronic schematics for the presented hardware interface to the 3M Bair Hugger 755. (PDF 84.1 kb) [file 12871_2018_542_MOESM1_ESM.pdf]
